# Whole transcriptome analysis of human erythropoietic cells during ontogenesis suggests a role of *VEGFA* gene as modulator of fetal hemoglobin and pharmacogenomic biomarker of treatment response to hydroxyurea in β-type hemoglobinopathy patients

**DOI:** 10.1186/s40246-017-0120-8

**Published:** 2017-10-23

**Authors:** Vasiliki Chondrou, Petros Kolovos, Argyro Sgourou, Alexandra Kourakli, Alexia Pavlidaki, Vlasia Kastrinou, Anne John, Argiris Symeonidis, Bassam R. Ali, Adamantia Papachatzopoulou, Theodora Katsila, George P. Patrinos

**Affiliations:** 1 Department of Pharmacy, School of Health Sciences, University of Patras, University Campus, Rion, GR-265 04 Patras, Greece; 2 Biotech Research and Innovation Centre (BRIC), University of Copenhagen, Copenhagen, Denmark; 3 Hellenic Open University, Patras, Greece; 4 Hematology Division, Department of Internal Medicine, Faculty of Medicine, University of Patras, Patras, Greece; 5 Department of Pathology, College of Medicine & Health Sciences, United Arab Emirates University, Al-Ain, United Arab Emirates; 6 Zayed Bin Sultan Center for Health Sciences, United Arab Emirates University, Al-Ain, United Arab Emirates; 7 Laboratory of General Biology, Faculty of Medicine, University of Patras, Patras, Greece; 8 Present address: Institut de Génétique et de Biologie Moléculaire et Cellulaire IGBMC/CNRS/INSERM/UDS, 67404 ILLKIRCH, BP 10142 CU de Strasbourg, France

**Keywords:** *VEGFA*, Beta-thalassemia, Transcriptomics, Ontogenesis, Sickle cell disease, Erythroid cell differentiation, Pharmacogenomics, Biomarkers

## Abstract

**Background:**

Human erythropoiesis is characterized by distinct gene expression profiles at various developmental stages. Previous studies suggest that fetal-to-adult hemoglobin switch is regulated by a complex mechanism, in which many key players still remain unknown. Here, we report our findings from whole transcriptome analysis of erythroid cells, isolated from erythroid tissues at various developmental stages in an effort to identify distinct molecular signatures of each erythroid tissue.

**Results:**

From our in-depth data analysis, pathway analysis, and text mining, we opted to focus on the *VEGFA* gene, given its gene expression characteristics. Selected *VEGFA* genomic variants, identified through linkage disequilibrium analysis, were explored further for their association with elevated fetal hemoglobin levels in β-type hemoglobinopathy patients. Our downstream analysis of non-transfusion-dependent β-thalassemia patients, β-thalassemia major patients, compound heterozygous sickle cell disease/β-thalassemia patients receiving hydroxyurea as fetal hemoglobin augmentation treatment, and non-thalassemic individuals indicated that *VEGFA* genomic variants were associated with disease severity in β-thalassemia patients and hydroxyurea treatment efficacy in SCD/β-thalassemia compound heterozygous patients.

**Conclusions:**

Our findings suggest that *VEGFA* may act as a modifier gene of human globin gene expression and, at the same time, serve as a genomic biomarker in β-type hemoglobinopathy disease severity and hydroxyurea treatment efficacy.

**Electronic supplementary material:**

The online version of this article (10.1186/s40246-017-0120-8) contains supplementary material, which is available to authorized users.

## Introduction

Hemoglobin is the key tetramer oxygen transport protein of red blood cells, composed of two pairs of identical globin chains (namely, the α-like and β-like chains). Different types of hemoglobin molecules are produced during embryonic, fetal, and adult erythropoiesis at different hematopoietic sites (yolk sac, fetal liver, and bone marrow, respectively) [34]. The differentiation of erythroid progenitors into mature erythrocytes as well as human globin expression needs to be strictly regulated aiming to maintain sufficient oxygen levels in all tissues [11, 20]. There are two developmental switches that occur upon the switch from primitive to definitive hematopoiesis and during the perinatal period. In the latter case, human fetal globin genes (*HBG1* and *HBG2*) that are expressed at high levels during the fetal stage are gradually downregulated, whereas human adult (*HBB* and *HBD*) globin genes are reciprocally upregulated. As a result, fetal hemoglobin (HbF) is expressed at high levels during fetal development and gradually declines to reach less than 2–3% of total hemoglobin levels shortly after birth.

Hemoglobinopathies are the most common single gene disorders and at the same time, one of the most devastating health problems worldwide, as their clinical phenotypes vary from mostly asymptomatic to severe anemia. Hemoglobinopathies are caused by quantitative (α- or β-thalassemia) or qualitative (hemoglobin variants and sickle cell disease, SCD) defects in hemoglobin production [5, 12]. Today, the most common therapeutic interventions for β-type hemoglobinopathies include regular blood transfusions, bone marrow transplantation, and transient pharmacological reactivation of HbF to compensate for the absent or deficient expression of adult hemoglobin. Hydroxyurea or hydroxycarbamide (HU) is the only FDA-approved drug for the treatment of SCD and β-thalassemia patients that transiently increases/reactivates HbF production and hence, ameliorates disease severity. However, HU is less effective in β-thalassemia than in SCD patients, while there is extreme inter-individual variability among β-type hemoglobinopathy patients in response to HU treatment [3, 23].

HbF production as well as the developmental switch from fetal to adult hemoglobin are regulated by various transcription factors and have been the focus of intensive investigations, since our understanding of the molecular mechanisms involved in fetal-to-adult globin gene switching would provide useful insights for β-type hemoglobinopathy therapeutics [19]. It has been proposed that there is a complex interplay between cis-acting elements within the human β-globin gene cluster and transcription factors, such as *MYB*, *BCL11A*, *KLF1* [39, 41], and others, that affect the rate of β-like globin gene transcription. Also, previous studies suggest that genomic loci residing outside the human β-globin gene cluster act as modifier genes to HbF production and are associated with elevated HbF levels and, as such, variable disease severity in β-type hemoglobinopathy patients and HU treatment response rate in SCD/β-thalassemia compound heterozygous patients [18, 25].

We have previously shown that genomic variants in the *MAP3K5*, *KLF10*, *SIN3A*, *NOS1*, *ARG1*, and *ARG2* genes are associated with elevated HbF levels and, hence, milder disease severity in β-type hemoglobinopathy patients and HU treatment response rate in SCD/β-thalassemia compound heterozygous patients [4, 7, 17, 40]. Herein, we adopted a whole transcriptome analysis of erythroid cells derived from human hematopoietic tissues of various developmental stages to identify candidate genes that are differentially expressed at various developmental stages. Following our data analysis coupled to pathway and linkage disequilibrium analyses, we show that selected *VEGFA* genomic variants are associated with β-thalassemia disease severity and HU treatment efficacy in SCD/β-thalassemia compound heterozygous patients, suggesting that *VEGFA* may be considered as a modifier gene for HbF production.

## Material and methods

### Subjects

For the purpose of microarray-based whole transcriptome analysis, we isolated and cultured erythroid progenitor cells from human fetal liver (*n* = 4), umbilical cord blood (*n* = 5), and adult peripheral blood (*n* = 4) samples, all from unrelated individuals of Caucasian origin. Human umbilical cord blood and fetal liver samples were collected and processed as described previously [24, 43, 44].

For tagSNP genotyping, we exploited a separate set of individuals, namely β-thalassemia major patients, non-transfusion-dependent thalassemia (NTDT) patients, SCD/β-thalassemia compound heterozygous patients, and ethnically matched healthy (non-thalassemic) individuals. β-thalassemia major patients differ from their NTDT counterparts in terms of their clinical phenotype and disease severity. In particular, β-thalassemia major patients require lifelong regular transfusions to survive and avoid disease complications, while NTDT patients present mild anemia (hemoglobin levels ranging from 7.7 to 11.2 g/dl) and need less frequent or no blood transfusions. The clinical phenotype of the NTDT patients included in this study cannot be attributed to α-globin gene variants (α-thalassemia) or hereditary persistence of fetal hemoglobin (HPFH syndrome). SCD/β-thalassemia compound heterozygous patients were systematically administered HU and characterized as “HU-responders” (plateau HbF levels above 20%) or “HU non-responders” (plateau HbF levels below 20%), based on their HbF expression levels following drug administration. All molecular, hematological, and clinical data of patient cohorts have been described in our previously published work [7, 15, 17, 32, 33, 40]. All tagSNP genotyping samples are of Hellenic origin, collected at the Patras University Hospital (Patras, Greece), AHEPA University Hospital (Thessaloniki, Greece), and Ippokrateio General Hospital of Thessaloniki (Thessaloniki, Greece).

### Whole transcriptome analysis

Microarray-based whole transcriptome analysis was performed as described previously [4]. In brief, erythroid progenitor cells from fetal liver, umbilical cord blood, and adult peripheral blood samples were isolated and cultured ex vivo. Total RNA was extracted, labeled, and hybridized to the Affymetrix Human Genome Array v2.0 according to manufacturer’s instructions (Affymetrix Inc., Santa Carla, CA, USA). Differential expression specifics were set by AltAnalyse. Probe sets were characterized and selected as significant, when their *p* value was < 0.05. Cluster 3.0 was used for data clustering of the short-listed probe sets [9] and JavaTree View Version 1.1.6r2 for data visualization [35]. A fold-change > 2 (FC > 2) is indicative of gene upregulation, whereas a fold-change < 2 (FC <2) corresponds to gene downregulation.

### TagSNP selection and linkage disequilibrium analysis

TagSNP selection across the *VEGFA* gene was carried out by the tagSNP picker program via the International HapMap Project (HapMap data release 27, phase II + III, February 2009, on NCBI36 assembly) and the LD TAG SNP Selection (tagSNP) (National Institute of Environmental Health Sciences) web-based tool. A tagged pairwise method was used with an *R*-square cutoff value of 0.8 and minor allele frequency (MAF) cutoff value of 0.2 [42, 45]. Pairwise linkage disequilibrium (LD) calculations were based on phase genotyped data (SNAP v2.2) [22], utilizing the HapMap Phase II+III (release 28) [2] and the 1000 Genomes Project (http://www.1000genomes.org) dataset for Caucasians (CEU). Findings were visualized on HaploView 4.2 and by using the LDmatrix module on LDlink web tool [26].

### Genotype analysis

Genomic DNA was extracted from whole blood leukocytes collected from healthy individuals and patients using the QIAamp Blood Kit (Qiagen GmbH, Hilden, Germany). Polymerase chain reaction (PCR) was carried out according to the KAPA2G Fast Hot Start protocol (KAPA Biosystems, MA, USA). A detailed description per tagSNP amplification conditions is available upon request. PCR products were purified with NucleoSpin Gel and PCR clean-up kit (Macherey-Nagel, GmbH, Düren, Germany) and subjected to direct DNA sequence analysis on an ABI Prim 3130xl DNA Analyser (Applied Biosystems) using the Big Dye^®^ Terminator v3.1 Cycle Sequencing Kit (Applied Biosystems, CA, USA), according to the manufacturer’s instructions.

### In silico analyses

Pathway analysis was performed with STRING software v9.1 (http://string-db.org). An emphasis was given to text mining and predicted interactions, both direct (physical) and indirect (functional). Venn diagrams were drawn with VENNY v2.1 (http://bioinfogp.cnb.csic.es/tools/venny/index.html). Gene ontology (GO) term analysis was performed with PANTHER classification system. Molecular function and biological process subcategories were subsequently identified [27]. For our downstream analysis, and to further investigate the role of the *VEGFA* tagSNPs in HU-mediated HbF production, we explored their effect on splicing motifs (including the “accept” and “donor” splice sites), the branch point and auxiliary sequences that enhance (exonic splicing enhancers, ESE) or repress (exonic splicing silencers, ESS) splicing. For this, in silico prediction took place, using Human Splicing Finder (http://www.umd.be/HSF3). This is a system that has quickly become an international reference, as it combines 12 algorithms [10].

### Statistical analysis

Hardy-Weinberg equilibrium was explored by Pearson’s goodness-of-fit chi-square (degree of freedom = 1), log-likelihood ratio chi-square (degree of freedom = 1), and exact test. De Finetti diagrams were also constructed [37]. Genotype and allele frequencies were evaluated using Fisher’s exact test. A two-tailed *p* value of < 0.05 was considered statistically significant. The *R* project for statistical computing (R i386 3.2.1) was used.

## Results

### Transcription profiling of human hematopoietic tissues during ontogenesis

Whole transcriptome analysis was performed to explore the transcription profiles of human hematopoietic tissues at different stages of ontogenesis. A unique molecular signature was obtained, consisting of genes that were differentially expressed among tissues that express (fetal liver, umbilical cord blood) HbF, do not express HbF, or marginally express (adult peripheral blood) HbF. Our comparative analysis includes three different datasets: (i) adult peripheral blood (low HbF) versus umbilical cord blood (high HbF), (ii) adult peripheral blood (low HbF) versus fetal liver (high HbF), and (iii) adult peripheral blood (low HbF) versus umbilical cord blood and fetal liver (high HbF).

**Fig. 1:**
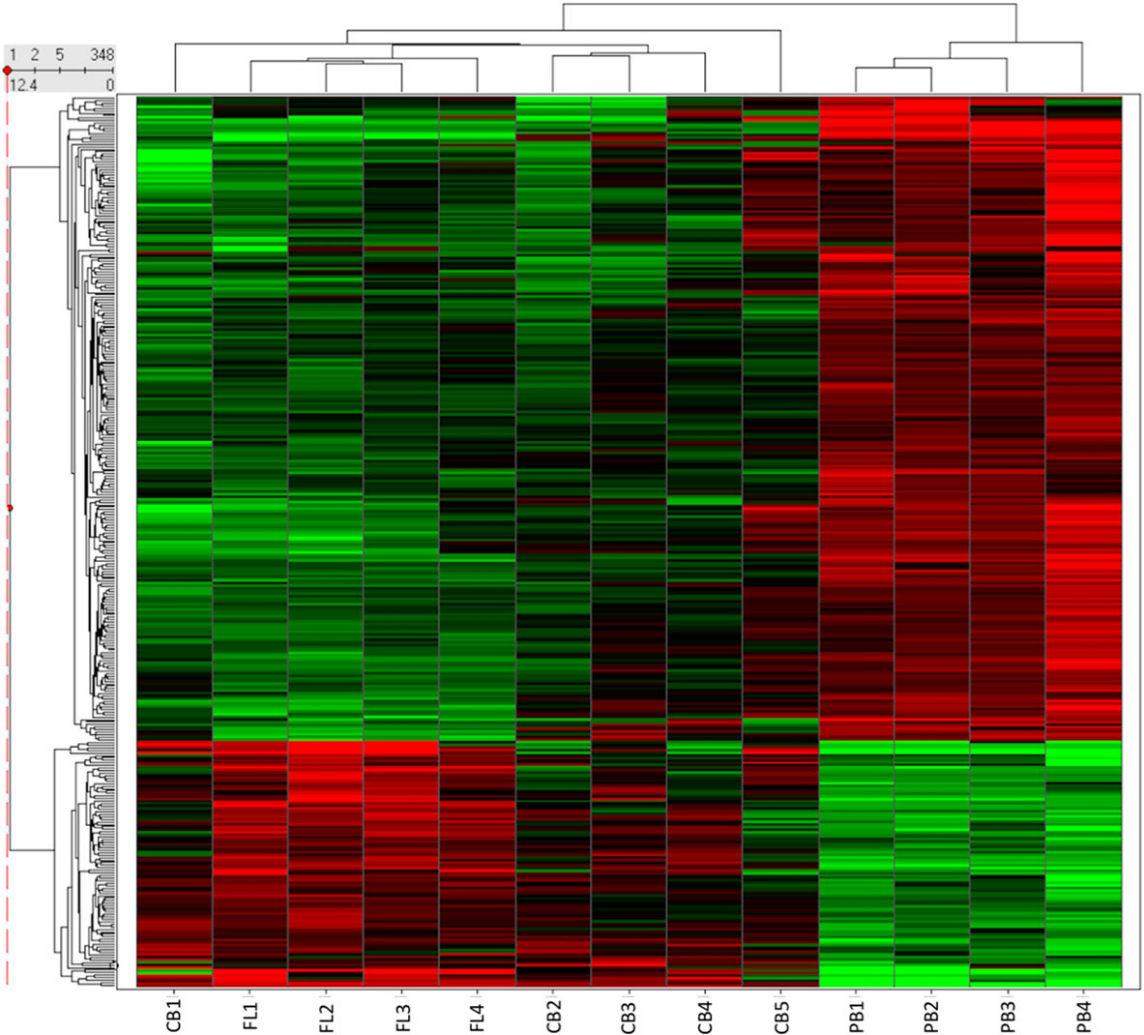
Differential gene expression when adult peripheral blood is compared to cord blood and fetal liver. A total of 264 genes were up- (FC > 2) or downregulated (FC < 2), when tissues with low HbF expression levels were compared to their counterparts with high HbF expression levels. Columns represent samples; rows are genes. Genes that were upregulated are depicted in red and genes that were downregulated are depicted in green

**Fig. 2:**
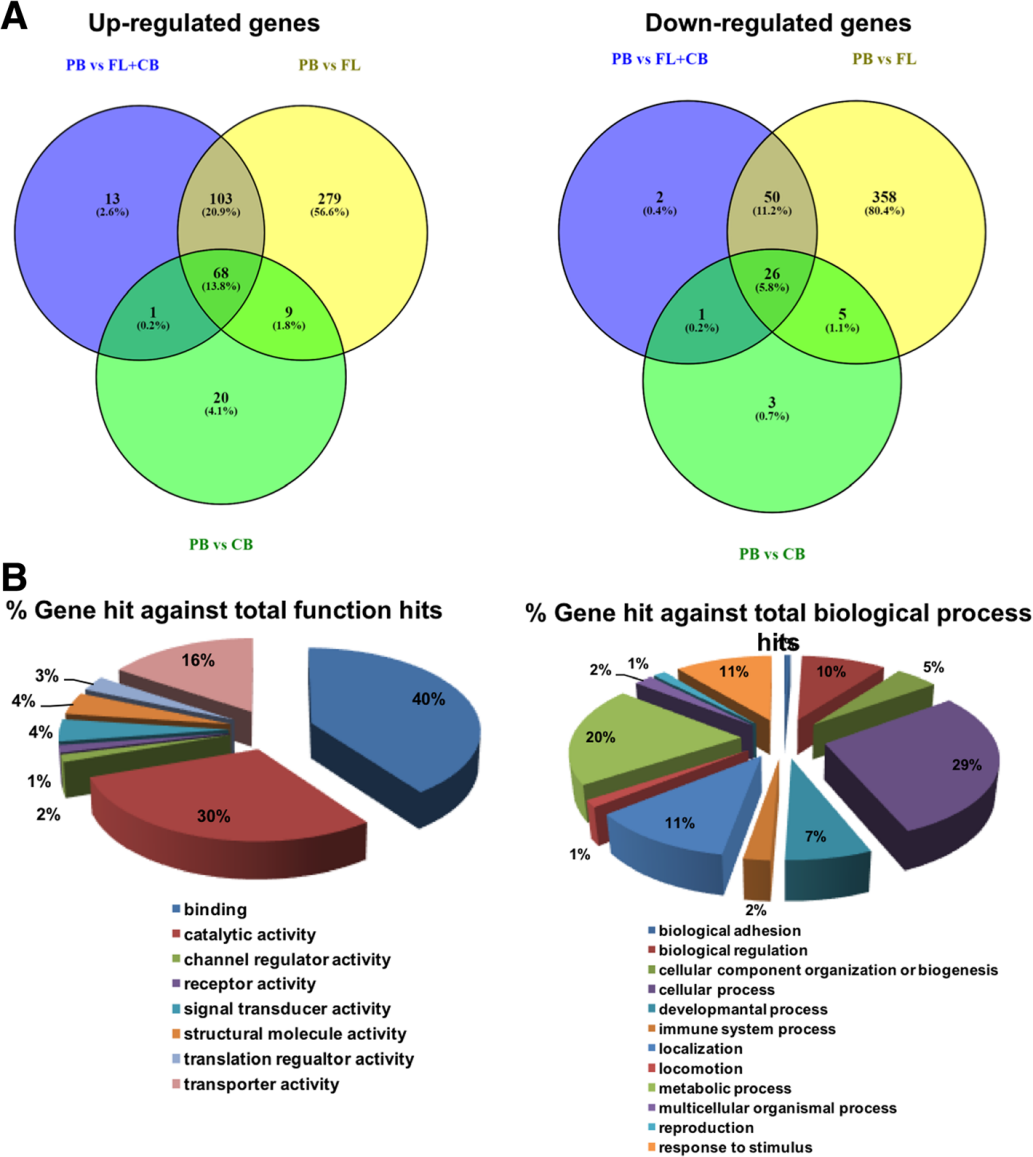
Common and unique up- and downregulated genes during ontogenesis. **a** The number in each circle represents the number of differentially expressed genes among the groups in question. Common and unique genes are shown. **b** PANTHER analysis outcomes (GO; molecular function and biological process)

When the first comparison was considered, a total of 165 probe sets representing 133 unique genes were differentially expressed (Additional file 1: Figure S1), while the second comparison revealed 1239 probe sets corresponding to 898 unique genes being differentially expressed (Additional file 1: Figure S2). When comparing all tissues (fetal liver and umbilical cord blood) that express high HbF levels versus peripheral blood that does not produce HbF, our analysis revealed 348 probe sets, corresponding to 264 unique genes that were differentially expressed among those tissues (Fig. 1). Data analysis revealed a candidate gene signature of erythropoiesis during human ontogenesis. Next, we focused on the comparison of the gene signature profile of those hematopoietic tissues, being most ontogenically distant, and thus, adult peripheral blood was compared to fetal liver (dataset ii) and/or adult peripheral blood was compared to umbilical cord blood and fetal liver (dataset iii). As indicated in Fig. 2 and Additional file 1: Tables S1 and S2, 103 genes were upregulated and 50 genes were downregulated, when datasets (ii) and (iii) were considered. Data were filtered on the basis of current publicly available literature and experimental evidence regarding gene-to-phenotype associations with β-hemoglobinopathies. We have subsequently performed GO term analysis. Pathway analysis and text mining were carried out for the genes included in the most dominant classifications of the GO term analysis. *VEGFA* was most prevalent in “Binding” (GO: 0005488), “Cellular process” (GO: 0009987), “Developmental process” (GO: 0002376) and “Response to stimulus” (GO: 0050896).

*VEGFA* was found to be upregulated (FC > 2) in adult peripheral blood (low HbF levels), when compared to umbilical cord blood and fetal liver (high HbF levels). *VEGFA* upregulation was even more profound, when adult peripheral blood was solely compared to fetal liver.

### TagSNPs across the *VEGFA* gene are associated with high HbF levels

**Fig. 3:**
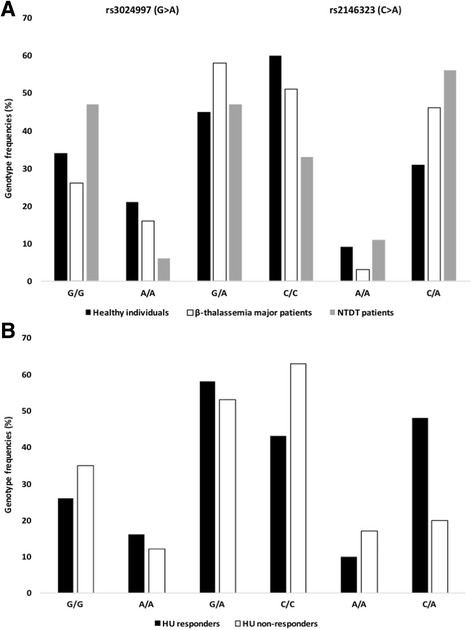
TagSNPs across the *VEGFA* gene are associated with disease phenotype (**a**) and HU treatment efficacy (**b**). **a** disease phenotype, rs3024997 (G>A; healthy individuals vs. NTDT patients *p* = 0.005; β-thalassemia major patients vs. NTDT patients *p* = 0.003) and rs2146323 (C>A; healthy individuals vs. β-thalassemia major patients *p* = 0.03; healthy individuals vs. NTDT patients *p* = 0.0005; β-thalassemia major patients vs. NTDT patients *p* = 0.009). **b** HU treatment efficacy, rs2146323 (C>A; HU responders vs. HU non-responders *p* = 0.0002). HU: hydroxyurea

Three tagSNPs (rs3024997, rs2146323, and rs10434) across the *VEGFA* gene were selected with MAF > 0.2% (CEU population) and explored further for their association with disease severity, attributed to high HbF levels. rs3024997 (G>A) and rs2146323 (C>A) are localized within intron 2, while rs10434 (A>G) within the 3′-untranslated region (3′-UTR) of the *VEGFA* gene. Genotyping data of β-type hemoglobinopathy patients and healthy (non-thalassemic) individuals of Hellenic origin are summarized in Table 1 and Additional file 1: Tables S3 and S4. A strong association for rs3024997 (G > A) with disease severity became evident, when NTDT patients (mild disease phenotype) and β-thalassemia major patients (severe disease phenotype) were compared (*p* = 0.003), and the same was true when NTDT patients versus non-thalassemic individuals (*p* = 0.005) were considered. Since the hοmozygous genotype for the rare allele (A) is less frequently found in NTDT patients, the presence of the A allele could be correlated with low HbF levels (Fig. 3a). rs2146323 (C > A) correlates well with disease severity and HbF levels, when (i) NTDT patients versus β-thalassemia major patients (*p* = 0.009), (ii) NTDT patients versus non-thalassemic individuals (*p* = 0.0005), and (iii) β-thalassemia major patients (*p* = 0.03) versus non-thalassemic individuals were considered. Our findings for the rare allele (A) suggest its association with high HbF levels (Fig. 3b). rs10434 (A > G) did not reach statistical significance, when genotype frequencies among patients and healthy individuals were compared.

**Table 1:** Summary of our genotype analysis of β-type hemoglobinopathy patients of Hellenic origin and healthy (non-thalassemic) individuals

Study population	Genotype frequency (%)								
	rs3024997 (G>A)			rs2146323 (C>A)			rs10434 (A>G)		
	G/G	A/A	G/A	C/C	A/A	C/A	A/A	G/G	A/G
Healthy individuals	34	21	45	60	9	31	18	31	51
β-thalassemia major patients	26	16	58	51	3	46	21	33	46
NTDT patients	47	6	47	33	11	56	21	29	50
HU responders	26	16	58	43	10	48	26	26	48
HU non-responders	35	12	53	63	17	20	25	29	46

The number of individuals per group (*n*) is indicated in parentheses per tagSNP: rs3024997 healthy individuals (*n* = 112), NTDT patients (*n* = 17), β-thalassemia major patients (n = 112), HU responders (*n* = 19), HU non-responders (*n* = 26); rs2146323 healthy individuals (*n* = 115), NTDT patients (*n* = 18), β-thalassemia major patients (*n* = 114), HU responders (*n* = 21), HU non-responders (*n* = 30); rs10434 healthy individuals (*n* = 72), NTDT patients (*n* = 14), β-thalassemia major patients (*n* = 105), HU responders (*n* = 19), HU non-responders (*n* = 28)

*HU* hydroxyurea, *NTDT* non-transfusion-dependent thalassemia

### TagSNPs across the *VEGFA* gene are associated with HU treatment efficacy in SCD/beta-thalassemia compound heterozygous patients

**Fig. 4:**
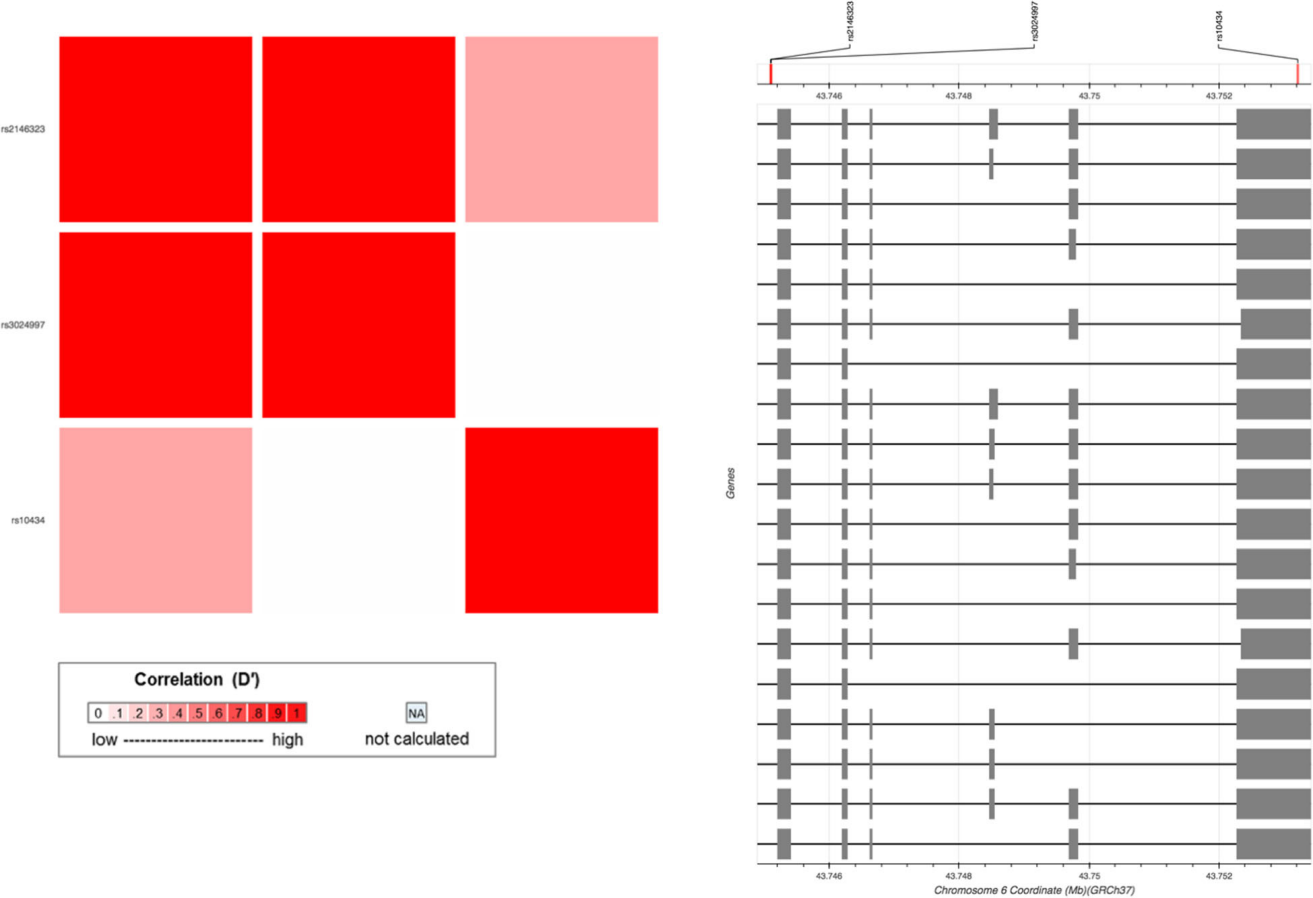
Pairwise linkage disequilibrium (LD) calculations for the tagSNPs of interest across the *VEGFA* gene (CEU). LD is measured as D′ and *R* ^2^ (see also Additional file 1: Table S2)

To explore if the selected tagSNPs across the *VEGFA* gene could serve as pharmacogenomic biomarkers, HU-responder and non-responder SCD/β-thalassemia patients were genotyped. A strong association for rs2146323 (C>A) with HU treatment efficacy (elevation of HbF levels) became evident (*p* = 0.0002), while neither of the rs3024997 (G>A) and rs10434 (A>G) tagSNPs reached statistical significance. As shown in Fig. 4 and Additional file 1: Table S4, pairwise LD calculations revealed that rs3024997 (G>A) and rs2146323 (C>A) are in complete LD (D′ = 1; *R* ^2^ < 1).

### In silico analyses

**Fig. 5:**
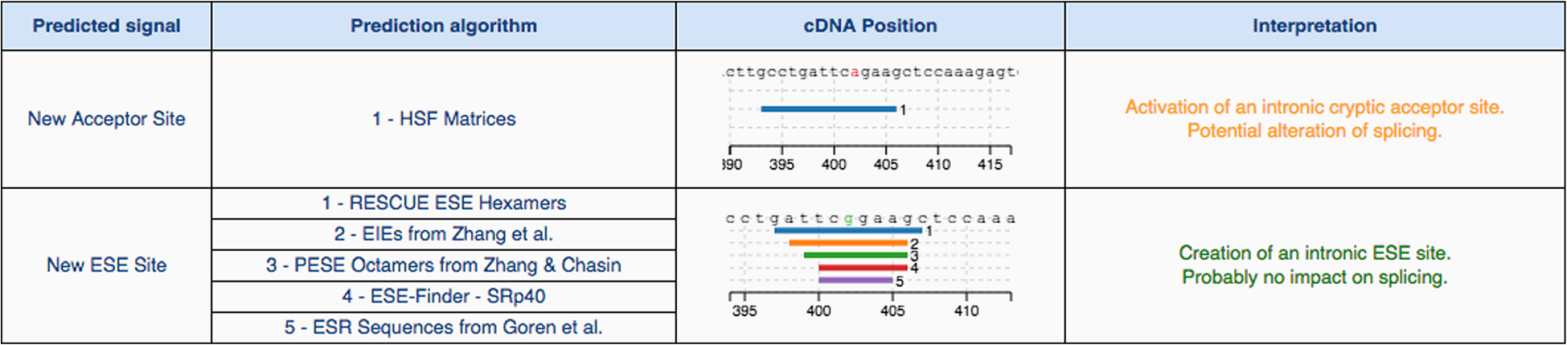
In silico analysis of the role of rs3024997 on splicing. For rs3024997 (G > A), the Human Splicing Finder prediction algorithm supports the activation of an intronic cryptic acceptor site and thus, potential alteration of splicing. Yet, the RESCUE ESE, EIEs, PESE Octamers, ESE Finder-SRp40, and ESR Sequences prediction algorithms suggest the creation of an exonic splicing enhancer, probably, with no impact on splicing. The graphic representation of the region confirms individual outcomes per algorithm considered, each one corresponding at a different color and length. For more information on the data, please visit http://www.umd.be/HSF3/technicaltips.html

According to the Human Splicing Finder prediction algorithm, rs3024997 (G>A) was found to occur in a deep intronic position, resulting on the activation of an intronic cryptic acceptor site and thus, potential alteration of splicing. Yet, the RESCUE ESE, EIEs, PESE Octamers, ESE Finder-SRp40, and ESR Sequences prediction algorithms suggest the creation of an exonic splicing enhancer, probably, with no impact on splicing (Fig. 5). rs2146323 (C>A) causes no significant splicing motif alteration, suggesting that the other co-inherited variants are possibly the key in its association with high HbF levels.

## Discussion

In humans, erythropoiesis as well as globin gene expression occurs at different sites during the various developmental stages in a time-specific manner (tissue- and development-specific expression). Hemoglobin switch consists of two separate events; the first event occurs early in endometrial life (embryonic to fetal hemoglobin switch), whereas the second one begins a few weeks before birth and ends ~ 6 months after delivery (fetal-to-adult hemoglobin switch). The understanding of the molecular mechanisms that govern hemoglobin switch holds a great promise for curing β-type hemoglobinopathies [34].

We have previously demonstrated that several genes, residing outside the human β-globin gene cluster, can be held responsible as genes modifying HbF levels in β-type hemoglobinopathy patients, reflecting on the disease severity and HU treatment response [4, 7, 18, 40]. Herein, we adopted a whole transcriptome analysis on tissues that express high and low HbF levels in an effort to identify a unique molecular signature corresponding to high HbF levels.

Following whole transcriptome and pathway analysis, coupled to text mining, we focused on the *VEGFA* gene since it shows a consistent gene expression pattern in most comparisons. We employed linkage disequilibrium analysis to select *VEGFA* genomic variants to further explore their association with elevated HbF levels in β-type hemoglobinopathy patients. In humans, *VEGFA* (belongs to the family of vascular endothelial growth factors, VEGFs) is localized on chromosome 6p21.1 (14 kb), consisting of 8 exons and 7 introns [21]. Studies in knockout mice indicate the role of *VEGFA* in the development of the vascular and hematopoietic tissues. Casella et al. [6] found that *VEGFA* production is crucial for the survival and differentiation of adult hematopoietic stem cells (HSCs), while *VEGFA* has been also shown to participate in hematopoiesis by an internal autocrine loop mechanism and be associated with adult *HBB* gene expression [13, 14]. Nakayama et al. [31] have shown that *VEGFA* synergizes with BMP-4 on the development of hematopoietic cells [31]. The same authors reported that murine embryonic stem cells were white, when grown only in the presence of BMP-4, becoming red, after the addition of *VEGFA* (and stem cell factor (SCF)). A few years later, Adelman et al. [1] showed that the BMP/BMPR/Smad pathway induces the expression of KLF1 and GATA-1 transcription factors during the differentiation of embryonic bodies in mice, yet β-globin expression is only detected, when SCF and *VEGFA* were present [1].

Consistent with this work, our previous findings indicate that at the late stages of human ontogenesis, where HbF levels are low, *VEGFA* mRNA levels are high. In particular, *VEGFA* is upregulated in cultured erythroid progenitor cells derived from adult peripheral blood (low HbF expression levels), when compared to progenitors derived from fetal liver or umbilical cord blood and fetal liver. Furthermore, *VEGFA* is downregulated in erythroblasts, when compared to early (BFU-Es) or late (CFU-Es) erythroid progenitors in hematopoietic tissues expressing high levels of HbF (umbilical cord blood and fetal liver, Kolovos P, unpublished). Such findings support further the inverse correlation between *VEGFA* and HbF levels that we observed during ontogenesis.

High HbF levels may improve disease severity in β-thalassemia and SCD, as β-globin deficiency is balanced by γ-globin synthesis and HbF prevents the polymerization of hemoglobin S, respectively [36]. Following the completion of the Human Genome Project, family and genome-wide association studies reported several SNPs that are associated with high HbF levels or a high proportion of F cells in β-type hemoglobinopathy patients, revealing a relatively large number of genetic modifiers of HbF expression [18, 19].

Today, although several compounds that transiently induce HbF levels (5-azacitidine, dexitabine) have been studied, HU is the only FDA-approved sickle cell disease and β-thalassemia treatment. HU is a cytotoxic antineoplastic agent that was initially administered to patients with myelodysplastic diseases and immune deficiencies as it is an inhibitor of ribonucleotide reductase, an enzyme necessary for DNA synthesis and repair. The drug mechanism towards the induction of HbF is not fully known. In β-thalassemia patients, HU administration leads to a 2- to a ~ 9-fold increase in γ-globin mRNA levels [30]. Inter-individual variability remains an issue and several studies concur that genomic variants residing in genes not only within, but also outside the human β-globin gene cluster, correlate with disease severity and HU treatment efficacy [18, 25].

Interestingly, our whole transcriptome analysis revealed that among those genes with a similar, to *VEGFA*, expression profile, there are genes previously described to be correlated with differentially produced HbF levels and with response to HU treatment in β-type hemoglobinopathy patients [7, 16, 25].

Following our whole transcriptome analysis, we focused on *VEGFA* gene variants and genotyped non-transfusion-dependent β-thalassemia patients, β-thalassemia major patients, and healthy (non-thalassemic) individuals as well as a cohort of compound heterozygous SCD/β-thalassemia patients receiving HU as HbF augmenting therapy. rs3024997 (G>A) was found to be strongly associated with low HbF levels and the severe phenotype of β-thalassemia major. A strong association was also observed between rs2146323 (C>A) and high HbF levels in NTDT patients and hence, a milder clinical phenotype of the disease. rs2146323 (C>A) may also serve as a pharmacogenomic biomarker for HU treatment efficacy. rs10434 (A>G) did not reach statistical significance. Small sample size remains a major limitation in all studies focusing on HU treatment individualization, including ours, especially for NTDT patients, due to the scarcity of such patients. Herein, Fisher’s exact test was chosen as the most appropriate test to guarantee type I error control, taking into account small sample sizes (Fisher’s exact test is considered more accurate than the chi-square test or G-test of independence in such cases). Yet, the lack of association for rs10434 due to small sample sizes cannot be excluded. *VEFGA* genomic variants have also been found in asymptomatic individuals with high HbF levels (unpublished), suggesting a possible role of *VEGFA* in increasing HbF levels.

According to the outcome of our in silico analyses, rs3024997 (G>A) was found to occur in a deep intronic position, resulting on the activation of an intronic cryptic acceptor site and thus, potential alteration of splicing. Yet, the RESCUE ESE, EIEs, PESE Octamers, ESE Finder-SRp40, and ESR Sequences prediction algorithms suggested the creation of an exonic splicing enhancer, which has probably no impact on splicing. rs2146323 (C>A) causes no significant splicing motif alteration, suggesting that the other co-inherited variants are possibly the key in its association with high HbF levels. No doubt, coupled to the bioinformatics predictions described, functional analysis needs to be performed, such as functional minigene analysis and exon-tiling microarrays to confirm the role of these variants as predicted in silico [8, 28, 29, 38].

Our study has a number of limitations. First of all, we have not managed to isolate fetal tissues from enough number of individuals to replicate our findings and this is subject to future work that is currently ongoing. Secondly, the sample size of the subsequent follow-up study was equally small, due to the lack of sufficient number of well-characterized β-thalassemia/SCD as well as NTDT patients, which are scarce and truly unique to find. Our results are, nevertheless, indicative for future replication work to validate the suggested role of *VEGFA* as possible modifier of HbF levels in adult life.

## Conclusion and future perspectives

Human hemoglobin switching still remains a great challenge, when β-type hemoglobinopathy management is considered. In a similar context, HbF production has attracted great interest over the last 50 years towards the amelioration of disease symptoms of β-type hemoglobinopathy patients. Taking into account that HU treatment efficacy exhibits great inter-individual variability, identification, and subsequently validation and clinical implementation of pharmacogenomic biomarkers hold promise for optimum patient stratification and disease management. Herein, our transcriptomics approach provided insights into the differential expression profiles of different erythroid tissues during human ontogenesis and suggested that *VEGFA* genomic variants serve both as genomic biomarkers for β-thalassemia disease severity and pharmacogenomic biomarkers for HU treatment efficacy in β-type hemoglobinopathy patients. In other words, a dual role is implied for *VEGFA* in erythropoiesis and HbF induction, which needs to be further investigated, if the molecular mechanism that delineate human hemoglobin switching is to be better defined and genome-based stratification of β-type hemoglobinopathy patients for HU treatment is to become reality. Notably, not all genomic loci that have been reported to increase HbF levels could be considered as pharmacogenomic biomarkers of HU treatment efficacy, as the clinical features in question, albeit related, are likely controlled by different molecular mechanisms. These findings, in conjunction to previous work conducted by our group and others, hold promise for the identification of pharmacogenomic biomarker for HbF-augmenting therapy, requiring carefully designed prospective clinical studies conducted by large multicenter consortia that include experienced clinicians and researchers from major clinical and academic centers.

## Additional file

Additional file 1: Table S1. Upregulated genes when (i) peripheral blood is compared to umbilical cord blood and fetal liver (PB vs CB+FL) and (ii) peripheral blood is compared to fetal liver (PB vs FL). **Table S2.** Downregulated genes when (i) peripheral blood is compared to umbilical cord blood and fetal liver (PB vs CB+FL) and (ii) peripheral blood is compared to fetal liver (PB vs FL). **Table S3.** Statistical and Hardy-Weinberg analyses on the genotyping data of β-type hemoglobinopathy patients of Hellenic origin and healthy (non-thalassemic) individuals. **Table S4.** Pairwise linkage disequilibrium (LD) calculations for the tagSNPs of interest across the *VEGFA* gene (CEU). **Figure S1.** Differential gene expression when adult peripheral blood is compared to cord blood. A total of 135 genes were over- (FC > 2) or downregulated (FC < 2). Columns represent samples; rows are genes. Red, genes that were upregulated; green, genes that were downregulated. **Figure S2.** Differential gene expression when adult peripheral blood is compared to fetal liver. A total of 962 genes were over- (FC > 2) or downregulated (FC < 2). Columns represent samples; rows are genes. Red, genes that were upregulated; green, genes that were downregulated. (PDF 822 kb)

## Abbreviations

CEU: Caucasian
HbF: Fetal hemoglobin
HPFH: Hereditary persistence of fetal hemoglobin
HU: Hydroxyurea
NTDT: Non-transfusion-dependent thalassemia
SCD: Sickle cell disease

## References

[1] Adelman CA, Chattopadhyay S, Bieker JJ (2002). The BMP/BMPR/Smad pathway directs expression of the erythroid-specific EKLF and GATA1 transcription factors during embryoid body differentiation in serum-free media. Development.

[2] Barrett JC, Fry B, Maller J, Daly MJ (2004). Haploview: analysis and visualization of LD and haplotype maps. Bioinformatics.

[3] Bordbar MR, Silavizadeh S, Haghpanah S, Kamfiroozi R, Bardestani M, Karimi M. Hydroxyurea treatment in transfusion-dependent β-thalassemia patients. Iran Red Crescent Med J. 2014;16:e18028. 10.5812/ircmj.18028 PMC4102988 25068055

[4] Borg J, Phylactides M, Bartsakoulia M, Tafrali C, Lederer C, Felice AE, Papachatzopoulou A, Kourakli A, Stavrou EF, Christou S (2012). KLF10 gene expression is associated with high fetal hemoglobin levels and with response to hydroxyurea treatment in β-hemoglobinopathy patients. Pharmacogenomics.

[5] Cao A, Galanello R (2010). Beta-thalassemia. Genet Med.

[6] Casella I, Feccia T, Chelucci C, Samoggia P, Castelli G, Guerriero R, Parolini I, Petrucci E, Pelosi E, Morsilli O (2003). Autocrine-paracrine VEGF loops potentiate the maturation of megakaryocytic precursors through Flt1 receptor. Blood.

[7] Chalikiopoulou C, Tavianatou A-G, Sgourou A, Kourakli A, Kelepouri D, Chrysanthakopoulou M, Kanelaki V-K, Mourdoukoutas E, Siamoglou S, John A (2016). Genomic variants in the ASS1 gene, involved in the nitric oxide biosynthesis and signaling pathway, predict hydroxyurea treatment efficacy in compound sickle cell disease/β-thalassemia patients. Pharmacogenomics.

[8] Cooper DN (2010). Functional intronic polymorphisms: buried treasure awaiting discovery within our genes. Human Genom.

[9] de Hoon MJ, Imoto S, Nolan J, Miyano S (2004). Open source clustering software. Bioinformatics.

[10] Desmet F-O, Hamroun D, Lalande M, Collod-Béroud G, Claustres M, Béroud C (2009). Human Splicing Finder: an online bioinformatics tool to predict splicing signals. Nucleic Acids Res.

[11] Dzierzak E, Philipsen S. Erythropoiesis: development and differentiation. Cold Spring Harbor Perspect Med. 2013;3:a011601. 10.1101/cshperspect.a011601 PMC3684002 23545573

[12] Galanello R, Origa R (2010). Beta-thalassemia. Orphanet J Rare Dis.

[13] Gerber H-P, Ferrara N (2003). The role of VEGF in normal and neoplastic hematopoiesis. J Mol Med.

[14] Gerber H-P, Malik AK, Solar GP, Sherman D (2002). VEGF regulates haematopoietic stem cell survival by an internal autocrine loop mechanism. Nature.

[15] Giannopoulou E, Bartsakoulia M, Tafrali C, Kourakli A, Poulas K, Stavrou EF, Papachatzopoulou A, Georgitsi M, Patrinos GP (2012). A single nucleotide polymorphism in the HBBP1 gene in the human β-globin locus is associated with a mild β-thalassemia disease phenotype. Hemoglobin.

[16] Giardine B, Borg J, Higgs DR, Peterson KR, Philipsen S, Maglott D, Singleton BK, Anstee DJ, Basak AN, Clark B (2011). Systematic documentation and analysis of human genetic variation in hemoglobinopathies using the microattribution approach. Nat Genet.

[17] Gravia A, Chondrou V, Kolliopoulou A, Kourakli A, John A, Symeonidis A, Ali BR, Sgourou A, Papachatzopoulou A, Katsila T (2016). Correlation of SIN3A genomic variants with β-hemoglobinopathies disease severity and hydroxyurea treatment efficacy. Pharmacogenomics.

[18] Gravia A, Chondrou V, Sgourou A, Papantoni I, Borg J, Katsila T, Papachatzopoulou A, Patrinos GP (2014). Individualizing fetal hemoglobin augmenting therapy for β-type hemoglobinopathies patients. Pharmacogenomics.

[19] Hagh MF, Fard AD, Saki N, Shahjahani M, Kaviani S (2011). Molecular mechanisms of hemoglobin F induction. Int J Hematol-Oncol Stem Cell Res.

[20] Hattangadi SM, Wong P, Zhang L, Flygare J, Lodish HF (2011). From stem cell to red cell: regulation of erythropoiesis at multiple levels by multiple proteins, RNAs, and chromatin modifications. Blood.

[21] Holmes DI, Zachary I (2005). The vascular endothelial growth factor (VEGF) family: angiogenic factors in health and disease. Genome Biol.

[22] Johnson, AD, Handsaker, RE, Pulit, SL, Nizzari, MM, O'donnell, CJ and De Bakker, PI. (2008). SNAP: a web-based tool for identification and annotation of proxy SNPs using HapMap. Bioinformatics 24, 2938-2939. 10.1093/bioinformatics/btn564 PMC2720775 18974171

[23] Koren A, Levin C, Dgany O, Kransnov T, Elhasid R, Zalman L, Palmor H, Tamary H (2008). Response to hydroxyurea therapy in β-thalassemia. Am J Hematol.

[24] Leberbauer C, Boulmé F, Unfried G, Huber J, Beug H, Müllner EW (2005). Different steroids co-regulate long-term expansion versus terminal differentiation in primary human erythroid progenitors. Blood.

[25] Ma Q, Wyszynski D, Farrell J, Kutlar A, Farrer L, Baldwin C, Steinberg M (2007). Fetal hemoglobin in sickle cell anemia: genetic determinants of response to hydroxyurea. Pharmacogenom J.

[26] Machiela MJ, Chanock SJ (2015). LDlink: a web-based application for exploring population-specific haplotype structure and linking correlated alleles of possible functional variants. Bioinformatics.

[27] Mi H, Muruganujan A, Casagrande JT, Thomas PD (2013). Large-scale gene function analysis with the PANTHER classification system. Nat Protocols.

[28] Millar DS, Horan M, Chuzhanova NA, Cooper DN (2010). Characterisation of a functional intronic polymorphism in the human growth hormone (GHI) gene. Human Genom.

[29] Moyer RA, Wang D, Papp AC, Smith RM, Duque L, Mash DC, Sadee W (2011). Intronic polymorphisms affecting alternative splicing of human dopamine D2 receptor are associated with cocaine abuse. Neuropsychopharmacology.

[30] Musallam KM, Taher AT, Cappellini MD, Sankaran VG (2013). Clinical experience with fetal hemoglobin induction therapy in patients with β-thalassemia. Blood.

[31] Nakayama N, Lee J, Chiu L (2000). Vascular endothelial growth factor synergistically enhances bone morphogenetic protein-4-dependent lymphohematopoietic cell generation from embryonic stem cells in vitro. Blood.

[32] Papachatzopoulou A, Kaimakis P, Pourfarzad F, Menounos PG, Evangelakou P, Kollia P, Grosveld FG, Patrinos GP (2007). Increased γ-globin gene expression in β-thalassemia intermedia patients correlates with a mutation in 3′ HS1. Am J Hematol.

[33] Papachatzopoulou A, Kourakli A, Makropoulou P, Kakagianne T, Sgourou A, Papadakis M, Athanassiadou A (2006). Genotypic heterogeneity and correlation to intergenic haplotype within high HbF β-thalassemia intermedia. Eur J Haematol.

[34] Patrinos GP, Antonarakis SE. Human hemoglobin. In: Vogel and Motulsky's human genetics. Vergal Berlin Heidelberg: Springer; 2010. p. 365–401.

[35] Saldanha AJ (2004). Java Treeview—extensible visualization of microarray data. Bioinformatics.

[36] Sankaran VG (2011). Targeted therapeutic strategies for fetal hemoglobin induction. ASH Educ Program Book.

[37] Sasieni PD. From genotypes to genes: doubling the sample size. Biometrics. 1997:1253–61. 9423247

[38] Seo S, Takayama K, Uno K, Ohi K, Hashimoto R, Nishizawa D, Ikeda K, Ozaki N, Nabeshima T, Miyamoto Y (2013). Functional analysis of deep intronic SNP rs13438494 in intron 24 of PCLO gene. PLoS One.

[39] Suzuki M, Yamamoto M, Engel JD (2014). Fetal globin gene repressors as drug targets for molecular therapies to treat the β-globinopathies. Mol Cell Biol.

[40] Tafrali C, Paizi A, Borg J, Radmilovic M, Bartsakoulia M, Giannopoulou E, Giannakopoulou O, Stojiljkovic-Petrovic M, Zukic B, Poulas K (2013). Genomic variation in the MAP3K5 gene is associated with β-thalassemia disease severity and hydroxyurea treatment efficacy. Pharmacogenomics.

[41] Tallack MR, Perkins AC (2013). Three fingers on the switch: Krüppel-like factor 1 regulation of γ-globin to β-globin gene switching. Curr Opin Hematol.

[42] Thorisson GA, Smith AV, Krishnan L, Stein LD (2005). The international HapMap project web site. Genome Res.

[43] Van Handel, B, Prashad, SL, Hassanzadeh-Kiabi, N, Huang, A, Magnusson, M, Atanassova, B, Chen, A, Hamalainen, EI and Mikkola, HK. (2010). The first trimester human placenta is a site for terminal maturation of primitive erythroid cells. Blood 116, 3321-3330. 10.1182/blood-2010-04-279489 PMC2995359 20628147

[44] Xu, J, Shao, Z, Glass, K, Bauer, DE, Pinello, L, Van Handel, B, Hou, S, Stamatoyannopoulos, JA, Mikkola, HK and Yuan, G-C. (2012). Combinatorial assembly of developmental stage-specific enhancers controls gene expression programs during human erythropoiesis. Dev Cell 23, 796-811. 10.1016/j.devcel.2012.09.003 PMC3477283 23041383

[45] Xu Z, Taylor JA (2009). SNPinfo: integrating GWAS and candidate gene information into functional SNP selection for genetic association studies. Nucleic Acids Res.

