# Supplementary material for: Whole transcriptome analysis of human erythropoietic cells during ontogenesis suggests a role of VEGFA gene as modulator of fetal hemoglobin and pharmacogenomic biomarker of treatment response to hydroxyurea in β-type hemoglobinopathy patients
Source: Hum Genomics. 2017 Oct 23;11:24. doi: 10.1186/s40246-017-0120-8 (PMC5654038; doi:10.1186/s40246-017-0120-8)

Supplementary Information

Whole transcriptome analysis of human erythropoietic cells during ontogenesis suggests a role of *VEGFA* gene as modulator of fetal hemoglobin and pharmacogenomic biomarker of treatment response to hydroxyurea in β-type hemoglobinopathies patients

Vasiliki Chondrou [1], Petros Kolovos [2], Argyro Sgourou [3], Alexandra Kourakli [4], Alexia Pavlidaki [1,%], Vlasia Kastrinou [1], Anne John [5], Argiris Symeonidis [4], Bassam R Ali [5,6], Adamantia Papachatzopoulou [7], Theodora Katsila [1], George P Patrinos [1,5,6,*]

[1] Department of Pharmacy, School of Health Sciences, University of Patras, Patras, Greece;

[2] Biotech Research and Innovation Centre (BRIC), University of Copenhagen, Copenhagen, Denmark;

[3] Hellenic Open University, Patras, Greece;

[4] Hematology Division, Department of Internal Medicine, Faculty of Medicine, University of Patras, Patras, Greece;

[5] Department of Pathology, College of Medicine & Health Sciences, United Arab Emirates University, Al-Ain, United Arab Emirates;

[6] Zayed Bin Sultan Center for Health Sciences, United Arab Emirates University, Al-Ain, United Arab Emirates;

[7] Laboratory of General Biology, Faculty of Medicine, University of Patras, Patras, Greece;

[%] Present address: Institut de Génétique et de Biologie Moléculaire et Cellulaire, IGBMC/CNRS/INSERM/UDS, BP 10142, 67404 ILLKIRCH, CU de Strasbourg, France

Supplementary Table 1: Up-regulated genes when (i) peripheral blood is compared to umbilical cord blood and fetal liver (PB vs CB+FL) and (ii) peripheral blood is compared to fetal liver (PB vs FL).

	**Gene**	**Fold change**	
		**PB vs CB+FL**	**PB vs FL**
1	Abhydrolase domain containing 5 *(ABHD5)*	2.622492275	3.158577955
2	Acyl-CoA synthetase long-chain family member 1 *(ACSL1)*	2.695445368	3.886746772
3	Adult retina protein *(LOC153222)*	3.836479082	3.802381808
4	Alanyl (membrane) aminopeptidase (aminopeptidase N, aminopeptidase M, microsomal aminopeptidase, CD13, p150) *(ANPEP)*	3.725120556	4.851373155
5	Aquaporin 1 (Colton blood group) *(AQP1)*	3.212601639	5.279028938
6	Aquaporin 3 (Gill blood group) *(AQP3)*	2.450076347	2.988984175
7	Arginase, type II *(ARG2)*	3.613212815	5.423182578
8	Argininosuccinate synthetase 1 *(ASS1)*	4.525992699	5.419764228
9	Brain expressed X-linked 2 *(BEX2)*	6.945779433	9.37818746
10	Calbindin 1, 28kDa *(CALB1)*	5.400211051	7.3433928
11	Caprin family member 2 *(CAPRIN2)*	2.410228563	7.299756216
12	Carbonic anhydrase I *(CA1)*	3.620053317	17.59825156
13	Carbonic anhydrase III, muscle specific *(CA3)*	6.832627049	10.97637993
14	CDNA clone IMAGE:2960540 *(IMAGE:2960540)*	3.157218537	3.637971722
15	CDNA clone IMAGE:4077090 *(IMAGE:4077090)*	3.501281265	4.085316904
16	CDNA FLJ13598 fis, clone PLACE1009921 *(FLJ13598 fis, clone PLACE1009921)*	4.384485484	8.48619052
17	CDNA FLJ38294 fis, clone FCBBF3010211 *(FCBBF3010211)*	3.705148914	3.576944373
18	CDNA FLJ39164 fis, clone OCBBF2002656 *(FLJ39164 fis, clone OCBBF2002656)*	4.082209084	5.334814127
19	Cerebellar degeneration-related protein 2, 62kDa *(CDR2)*	3.023791176	3.504359915
20	Chemokine (C-C motif) receptor-like 2 /// similar to chemokine (C-C motif) receptor-like 2 *(CCRL2)*	2.797503169	3.05214658
21	Chloride intracellular channel 2 *(CLIC2)*	3.708613174	7.807914676
22	Chromobox homolog 4 (Pc class homolog, Drosophila) *(CBX4)*	2.412019839	3.330428652
23	Chromosome 16 open reading frame 45 *(c16orf45)*	3.094423866	3.641006517

	Gene	Fold change	
		PB vs CB+FL	PB vs FL
24	Chromosome 18 open reading frame 54 *(c18orf54)*	2.698998863	2.900329498
25	Chromosome 6 open reading frame 85 *(c6orf85)*	2.833636218	4.116658639
26	Collagen, type XV, alpha 1 *(COL15A1)*	3.360363394	5.631413627
27	C-type lectin domain family 2, member B *(CLEC2B)*	4.780749708	5.134772508
28	Cyclin E2 *(CCNE2)*	2.630337519	3.563666808
29	Cyclin G2 *(CCNG2)*	4.310290094	5.003342199
30	Cytoplasmic polyadenylation element binding protein 4 *(CPEB4)*	3.220709299	2.997618179
31	Death-associated protein kinase 1 *(DAPK1)*	3.418453562	4.562000202
32	Erythrocyte membrane protein band 4.1 like 4A *(EPB41L4A)*	3.044804445	3.031319356
33	Eukaryotic translation initiation factor 4B *(EIF4B)*	2.477766535	2.331434714
34	Family with sequence similarity 129, member A *(FAM129A)*	3.938814688	5.929304871
35	Family with sequence similarity 19 (chemokine (C-C motif)-like), member A2 *(FAM19A2)*	3.007321258	5.021566613
36	FLJ41603 protein *(FLJ41603 protein)*	3.047502752	5.439389845
37	Forkhead box O1 *(FOXO1)*	5.544990988	4.820842258
38	Forkhead box O3 *(FOXO3)*	3.298149808	3.940756072
39	Full length insert cDNA clone ZE01A04 *(ZE01A04)*	3.546099103	9.296870957
40	Glutamine-fructose-6-phosphate transaminase 1 *(GFPT1)*	2.79883562	3.347817348
41	Glutamyl-prolyl-tRNA synthetase *(EPRS)*	2.460069419	2.759411242
42	Grainyhead-like 1 (Drosophila) *(GRHL1)*	5.199061764	6.846860186
43	GRB2-associated binding protein 2 *(GAB2)*	3.776169216	3.652174464
44	Growth differentiation factor 15 *(GDF15)*	3.39635034	7.552139574
45	GTP cyclohydrolase 1 (dopa-responsive dystonia) *(GCH1)*	4.809902234	10.7000921
46	Guanine nucleotide binding protein (G protein), alpha 13*(GNA13)*	3.238156457	5.142374595
47	Headcase homolog (Drosophila) *(HECA)*	2.836070928	3.181720263
48	Hemoglobin, delta *(HBD)*	2.939071082	6.742514671
49	Homeodomain interacting protein kinase 2 *(HIPK2)*	2.80259823	3.184614641
50	Hypothetical protein FLJ20309 *(FLJ20309)*	3.848681707	5.224824329
51	Hypothetical protein LOC728177 *(LOC728177)*	3.838535738	5.726743805
52	Hypothetical protein MGC14376 *(MGC14376)*	3.458267787	5.718472644

	Gene	Fold change	
		PB vs CB+FL	PB vs FL
53	Insulin receptor substrate 2 *(IRS2)*	2.761324541	3.292273215
54	Interleukin 6 signal transducer (gp130, oncostatin M receptor) *(IL6ST)*	3.287644588	2.897845459
55	Interleukin 8 *(IL8)*	6.746065908	10.40248731
56	JAZF zinc finger 1 *(JAZF1)*	2.704674137	5.563255842
57	Jumonji C domain-containing histone demethylase 1 homolog D (S. cerevisiae) *(JHDM1D)*	2.868499777	3.284062335
58	Kelch domain containing 8A *(KLHDC8A)*	3.503081802	5.791548778
59	KIAA0746 protein (*KIAA0746)*	2.682592129	2.886614058
60	KIAA0774 *(KIAA0774)*	2.874881968	5.351106442
61	KIAA1727 protein *(KIAA1727)*	2.759577804	3.240812329
62	Kruppel-like factor 9 *(KLF9)*	3.408182912	4.003078211
63	MICAL-like 1 *(MICALL1)*	2.757589995	4.808485675
64	Monocyte to macrophage differentiation-associated *(MMD)*	3.161148132	4.945639412
65	Motile sperm domain containing 1 *(MOSPD1)*	2.808750295	3.337524136
66	MSTP150 *(MST150)*	2.593963977	2.593956831
67	Muscleblind-like 2 (Drosophila) *(MBNL2)*	4.476080688	5.310217705
68	Neogenin homolog 1 (chicken) *(NEO1)*	2.476856454	2.681794982
69	Neural precursor cell expressed, developmentally down-regulated 4-like *(NEDD4L)*	2.556918985	3.75247555
70	Nidogen 2 (osteonidogen) *(NID2)*	2.796979287	3.230120351
71	Nuclear receptor coactivator 7 *(NCOA7)*	2.371514125	2.436608525
72	Nudix (nucleoside diphosphate linked moiety X)-type motif 4 *(NUDT4)*	2.991027407	3.362924845
73	Oligodendrocyte transcription factor 1 *(OLIG1)*	2.822179162	6.740682544
74	Optineurin *(OPTN)*	3.246567573	7.741918775
75	PDZK1 interacting protein 1 *(PDZK1P1)*	5.838845716	11.02727389
76	Phospholipid scramblase 4 *(PLSCR4)*	3.100107165	5.856862638
77	Progestin and adipoQ receptor family member IX *(PAQR9)*	3.807640125	7.150067767
78	Proline rich Gla (G-carboxyglutamic acid) 4 (transmembrane) *(PRRG4)*	3.374160284	4.30318811
79	RAB6B, member RAS oncogene family *(RAB6B)*	2.535763302	3.77261755
80	Ras-like without CAAX 1 *(RIT1)*	2.534180806	2.265072235

	Gene	Fold change	
		PB vs CB+FL	PB vs FL
81	Ring finger protein (C3H2C3 type) 6 *(RNF6)*	2.519394226	2.834726088
82	Ring finger protein 213 *(RNF213)*	2.398372023	2.509886633
83	Scm-like with four mbt domains 2 *(SFMBT2)*	2.894558285	2.545283006
84	SCY1-like 2 (S. cerevisiae) *(SCYL2)*	2.491513617	3.074480026
85	SEC14-like 1 (S. cerevisiae) *(SEC14A1)*	2.924001648	3.355409615
86	SEC14-like 2 (S. cerevisiae) *(SEC14L2)*	2.566521509	2.408693143
87	Similar to CG32820-PA, isoform A *(LOC653071)*	2.467109532	2.185568192
88	SLIT-ROBO Rho GTPase activating protein 2 pseudogene 1*(SRGAP2P1)*	3.246159377	3.704836543
89	Solute carrier family 22 (organic cation transporter), member 16 *(SLC22A16)*	2.863596669	9.112250157
90	Solute carrier family 30 (zinc transporter), member 1 *(SLC30A1)*	2.674524865	4.819601532
91	Solute carrier family 7, (cationic amino acid transporter, y+ system) member 11 *(SLC7A11)*	3.498194845	4.7423717
92	Stress 70 protein chaperone, microsome-associated, 60kDa *(STCH)*	3.043457333	2.937887473
93	Thymic stromal lymphopoietin *(TSLP)*	4.022564974	4.266251632
94	Thyroid hormone receptor, beta (erythroblastic leukemia viral (v-erb-a) oncogene homolog 2, avian) *(THRB)*	2.706445116	8.228057246
95	Transferrin receptor (p90, CD71) *(TFRC)*	3.153930531	5.447275579
96	Tribbles homolog 3 (Drosophila) *(TRIB3)*	3.229472905	4.740963901
97	Tripartite motif-containing 10 *(TRIM10)*	2.597688815	5.049041674
98	Tubulin, alpha 1a *(TUBA1A)*	2.927181796	3.613938904
99	Tubulin, gamma 2 *(TUBG2)*	3.357138272	7.044498648
100	Tuftelin 1 *(TUFT1)*	4.166335948	5.159493126
101	Ubiquitin specific peptidase 34 *(USP34)*	2.513158144	2.812951051
102	Vascular endothelial growth factor A *(VEGFA)*	2.889604144	4.377167706
103	WWC family member 3 *(WWC3)*	2.672864613	3.997286684

Supplementary Table 2: Down-regulated genes when (i) peripheral blood is compared to umbilical cord blood and fetal liver (PB vs CB+FL) and (ii) peripheral blood is compared to fetal liver (PB vs FL).

	**Gene**	**Fold change**	
		**PB vs CB+FL**	**PB vs FL**
1	Adenosine deaminase *(ADA)*	0.395281897	0.393152952
2	Amphiregulin (schwannoma-derived growth factor) /// similar to Amphiregulin precursor (AR) (Colorectum cell-derived growth factor) (CRDGF) *(AREG /// LOC727738)*	0.1157324	0.064626564
3	Cat eye syndrome chromosome region, candidate 5 *(CECR5)*	0.337149036	0.261849844
4	Cathepsin H *(CTSH)*	0.317014424	0.284292471
5	CCAAT/enhancer binding protein (C/EBP), delta *(CEBPD)*	0.276370643	0.200782683
6	Cell division cycle associated 7 *(CDCA7)*	0.324578476	0.262878583
7	Chromosome 10 open reading frame 58 *(C10orf58)*	0.250114554	0.199360178
8	Clone DNA57836 GLPG464 *(UNQ464)*	0.221398963	0.252663338
9	Collagen, type IV, alpha 5 (Alport syndrome) *(COL4A5)*	0.189214953	0.167374241
10	CXXC finger 6 *(CXXC6)*	0.137914589	0.096035936
11	Cysteine-rich secretory protein 2 *(CRISP2)*	0.211677919	0.159079366
12	Estrogen-related receptor gamma *(ESRRG)*	0.099543565	0.083323769
13	Forkhead box D1 *(FOXD1)*	0.177940302	0.110259099
14	GATA binding protein 5 *(GATA5)*	0.155648027	0.364965985
15	Glucose phosphate isomerase *(GPI)*	0.326384891	0.341577522
16	Glycine amidinotransferase (L-arginine:glycine amidinotransferase) *(GATM)*	0.172409813	0.118386675
17	Guanine nucleotide binding protein (G protein), alpha inhibiting activity polypeptide 1 *(GNAI1)*	0.237385011	0.255466082
18	H19, imprinted maternally expressed untranslated mRNA *(H19)*	0.046728474	0.027819461
19	Hemoglobin, zeta *(HBZ)*	0.052191081	0.032028306
20	Hexokinase 2 *(HK2)*	0.273909443	0.177934739
21	Homo sapiens, clone IMAGE:5189615, mRNA	0.199374192	0.375102244
22	Hypermethylated in cancer 2 *(HIC2)*	0.337638106	0.309972074
23	Hypothetical protein LOC51252 *(LOC51252)*	0.207826069	0.243203916

	Gene	Fold change	
		PB vs CB+FL	PB vs FL
24	Insulin-like growth factor binding protein 7 *(IGFBP7)*	0.213889547	0.135574226
25	Kelch-like 23 (Drosophila) *(KLHL23)*	0.318729134	0.248003805
26	Kinase suppressor of ras 1 *(KSR1)*	0.151386942	0.10243736
27	Lin-28 homolog B (C. elegans) *(LIN28B)*	0.034141802	0.017967316
28	Lysosomal associated protein transmembrane 4 beta*(LAPTM4B)*	0.259147404	0.191860343
29	Lysozyme (renal amyloidosis) *(LYZ)*	0.117876616	0.065114873
30	Myeloperoxidase *(MPO)*	0.058082297	0.039551912
31	Myocyte enhancer factor 2C *(MEF2C)*	0.375143164	0.289609144
32	Myo-inositol 1-phosphate synthase A1 *(ISYNA1)*	0.255979408	0.180255561
33	Neurofilament, heavy polypeptide 200kDa *(NEFH)*	0.178112476	0.181318527
34	Paroxysmal nonkinesigenic dyskinesia *(PNKD)*	0.160342134	0.148617
35	Phospholipid transfer protein *(PLTP)*	0.251521644	0.217698982
36	Prostaglandin E receptor 3 (subtype EP3) *(PTGER3)*	0.169488446	0.109321197
37	Protein kinase D3 *(PRKD3)*	0.169890314	0.21144358
38	Protein phosphatase 1, regulatory (inhibitor) subunit 3C *(PPP1R3C)*	0.151233014	0.091320948
39	Ribonuclease T2 *(RNASET2)*	0.320412286	0.243865939
40	Roundabout, axon guidance receptor, homolog 1 (Drosophila) *(ROBO1)*	0.164244223	0.128566225
41	Sema domain, immunoglobulin domain (Ig), transmembrane domain (TM) and short cytoplasmic domain, (semaphorin) 4C*(SEMA4C)*	0.348320336	0.345862429
42	Sorbitol dehydrogenase *(SORD)*	0.222819425	0.247597621
43	TIMP metallopeptidase inhibitor 3 (Sorsby fundus dystrophy, pseudoinflammatory) *(TIMP3)*	0.246533176	0.200699425
44	Tribbles homolog 2 (Drosophila) *(TRIB2)*	0.213758187	0.256114274
45	Tripartite motif-containing 6 *(TRIM6)*	0.286319953	0.263724872
46	TSPY-like 5 *(TSPYL5)*	0.278689444	0.192915013
47	Tubulin, beta 1 *(TUBB1)*	0.240015322	0.232022434
48	Von Willebrand factor C and EGF domains *(VWCE)*	0.282196072	0.196505457
49	Wingless-type MMTV integration site family, member 5B *(WNT5B)*	0.129656711	0.274701149
50	Zinc finger CCCH-type, antiviral 1-like *(ZC3HAV1L)*	0.326617199	0.241993625

Supplementary Table 3. Statistical and Hardy-Weinberg analyses on the genotyping data of β-type hemoglobinopathies patients of Hellenic origin and healthy (non-thalassemic) individuals.

**Study population**	**rs3024997 (G>A)**	**rs2146323 (C>A)**	**rs2146323 (C>A)**
	**Statistical analysis (p-values)**		
Healthy individuals vs. β-thalassemia major patients	0.199	0.03	PA=0.748; PB=0.735
	**Hardy-Weinberg analysis (p-values)**		
	Controls<br>0.397952 (Pearson)<br>0.398091 (Llr)<br>0.418000 (Exact)<br>Cases<br>0.085948 (Pearson)<br>0.084735 (Llr)<br>0.108678 (Exact)	Controls<br>0.105127 (Pearson)<br>0.115340 (Llr)<br>0.107909 (Exact)<br>Cases<br>0.050670 (Pearson)<br>0.038290 (Llr)<br>0.069013 (Exact)	Controls<br>0.707405 (Pearson)<br>0.707247 (Llr)<br>0.839094 (Exact)<br>Cases<br>p=0.505677 (Pearson)<br>p=0.505778 (Llr)<br>p=0.543615 (Exact)
Healthy individuals vs. NTDT patients	**Statistical analysis (p-values)**		
	0.005	0.0005	0.896
	**Hardy-Weinberg analysis (p-values)**		
	Controls<br>0.397952 (Pearson)<br>0.398091 (Llr)<br>0.418000 (Exact)<br>Cases<br>0.193795 (Pearson)<br>0.183849 (Llr)<br>0.237330 (Exact)	Controls<br>0.105127 (Pearson)<br>0.115340 (Llr)<br>0.107909 (Exact)<br>Cases<br>0.076785 (Pearson)<br>0.073850 (Llr)<br>0.095905 (Exact)	Controls<br>0.707405 (Pearson)<br>0.707247 (Llr)<br>0.839094 (Exact)<br>Cases<br>0.948642 (Pearson)<br>0.948640 (Llr)<br>1.000000 (Exact)
β-Thalassemia major patients vs. NTDT patients	**Statistical analysis (p-values)**		
	0.003	0.009	0.823
	**Hardy-Weinberg analysis (p-values)**		
	Controls<br>0.085948 (Pearson)<br>0.084735 (Llr)<br>0.108678 (Exact)<br>Cases<br>0.193795 (Pearson)<br>0.183849 (Llr)<br>0.237330 (Exact)	Controls<br>0.050670 (Pearson)<br>0.038290 (Llr)<br>0.069013 (Exact)<br>Cases<br>0.076785 (Pearson)<br>0.073850 (Llr)<br>0.095905 (Exact)	Controls<br>0.505677 (Pearson)<br>0.505778 (Llr)<br>0.543615 (Exact)<br>Cases<br>0.948642 (Pearson)<br>0.948640 (Llr)<br>1.000000 (Exact)
HU responders vs. HU non-responders	**Statistical analysis (p-values)**		
	0.337	0.0002	PA=0.932; PB=0.917
	**Hardy-Weinberg analysis (p-values)**		
	Controls<br>0.085948 (Pearson)<br>0.084735 (Llr)<br>0.108678 (Exact)<br>Cases<br>0.233238 (Pearson)<br>0.230303 (Llr)<br>0.293727 (Exact)	Controls<br>0.519504 (Pearson)<br>0.517052 (Llr)<br>0.656613 (Exact)<br>Cases<br>8.374e-07 (Pearson)<br>1.857e-06 (Llr)<br>2.134e-06 (Exact)	Controls<br>p=0.689157 (Pearson)<br>p=0.689117 (Llr)<br>p=0.691924 (Exact)<br>Cases<br>p=0.432303 (Pearson)<br>p=0.432102 (Llr)<br>p=0.428313 (Exact)

(Pearson): Pearson's goodness-of-fit chi-square (degree of freedom=1); (Llr): Log likelihood ratio chi-square (degree of freedom=1); (Exact): Exact test PA, PB: two-tailed p-values; if P, then PA=PB. Data shown in red correspond to significant p-values (p-values < 0.05) for the statistical test noted in the parentheses.

Supplementary Table 4. Pair-wise linkage disequilibrium (LD) calculations for the tagSNPs of interest across the *VEGFA* gene (CEU).

**D'**	**rs3024997 (G>A)**	**rs2146323 (C>A)**	**rs10434 (A>G)**
**rs3024997 (G>A)**	1.0	1.0	0.009
**rs2146323 (C>A)**	1.0	1.0	0.349
**rs10434 (A>G)**	0.009	0.349	1.0
**R^2**	**rs3024997 (G>A)**	**rs2146323 (C>A)**	**rs10434 (A>G)**
**rs3024997 (G>A)**	1.0	0.244	0.0
**rs2146323 (C>A)**	0.244	1.0	0.044
**rs10434 (A>G)**	0.0	0.044	1.0

Supplementary Figures

Supplementary Figure 1.

Differential gene expression when adult peripheral blood is compared to cord blood. A total of 135 genes were over- (FC >2) or down-regulated (FC <2). Columns represent samples; rows are genes. Red, genes that were up-regulated; green, genes that were down-regulated.

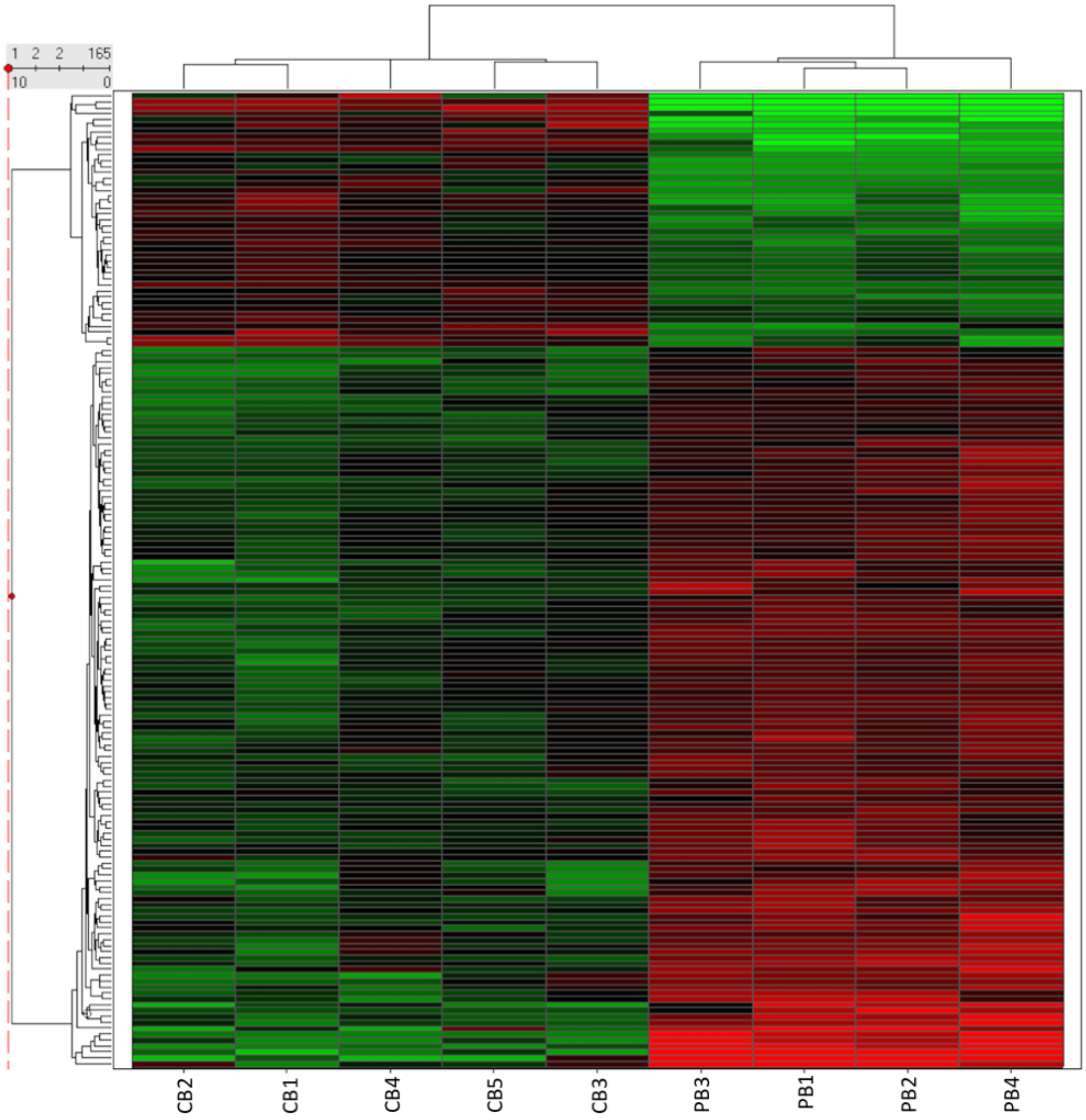

Supplementary Figure 2.

Differential gene expression when adult peripheral blood is compared to fetal liver. A total of 962 genes were over- (FC >2) or down-regulated (FC <2). Columns represent samples; rows are genes. Red, genes that were up-regulated; green, genes that were down-regulated.

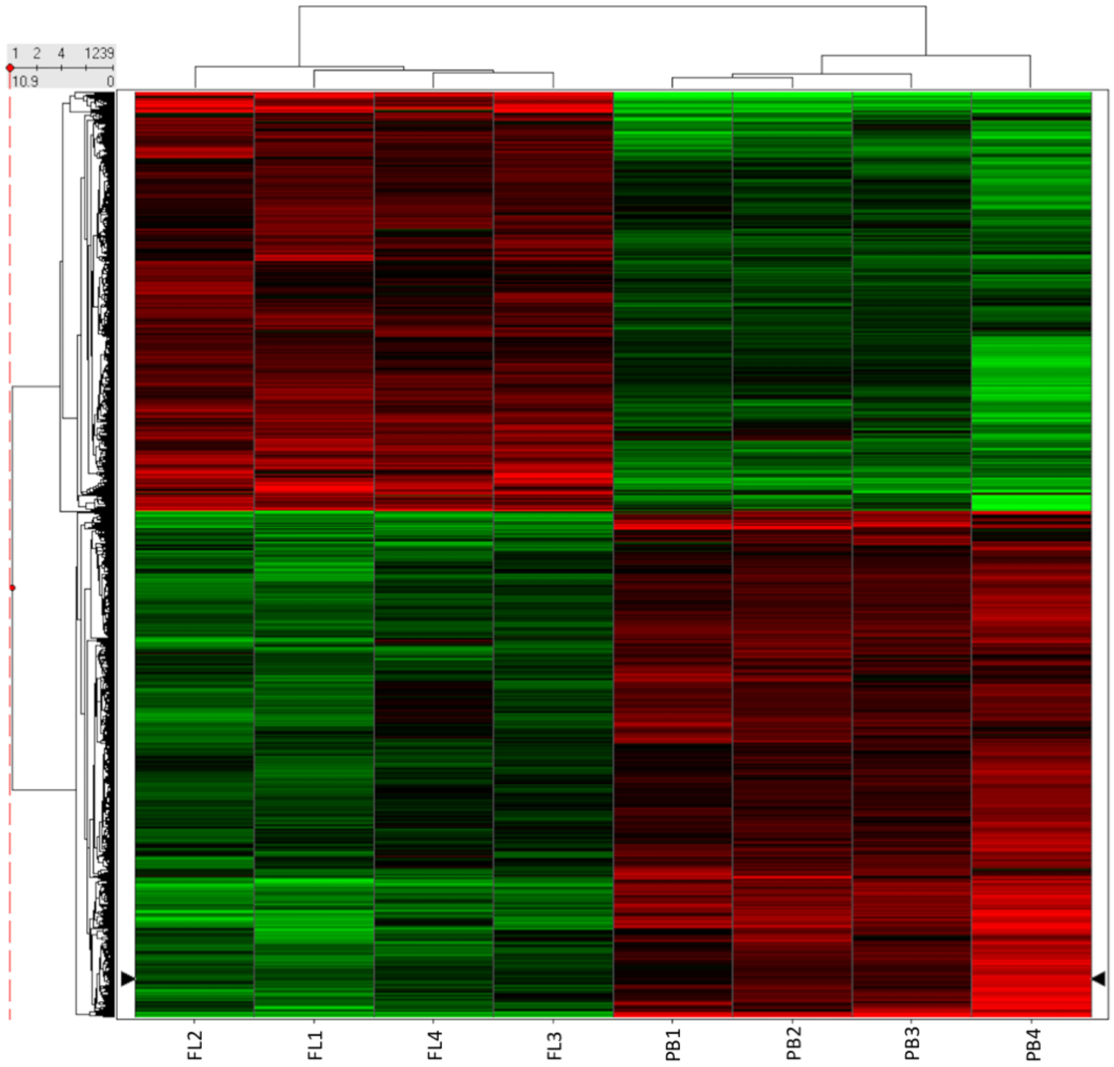

Supplement: Additional file 1: Table S1. — Upregulated genes when (i) peripheral blood is compared to umbilical cord blood and fetal liver (PB vs CB+FL) and (ii) peripheral blood is compared to fetal liver (PB vs FL). Table S2. Downregulated genes when (i) peripheral blood is compared to umbilical cord blood and fetal liver (PB vs CB+FL) and (ii) peripheral blood is compared to fetal liver (PB vs FL). Table S3. Statistical and Hardy-Weinberg analyses on the genotyping data of β-type hemoglobinopathy patients of Hellenic origin and healthy (non-thalassemic) individuals. Table S4. Pairwise linkage disequilibrium (LD) calculations for the tagSNPs of interest across the VEGFA gene (CEU). Figure S1. Differential gene expression when adult peripheral blood is compared to cord blood. A total of 135 genes were over- (FC > 2) or downregulated (FC < 2). Columns represent samples; rows are genes. Red, genes that were upregulated; green, genes that were downregulated. Figure S2. Differential gene expression when adult peripheral blood is compared to fetal liver. A total of 962 genes were over- (FC > 2) or downregulated (FC < 2). Columns represent samples; rows are genes. Red, genes that were upregulated; green, genes that were downregulated. (PDF 822 kb) [file 40246_2017_120_MOESM1_ESM.pdf]
